# Complete Genome Sequence of an Enterovirus A71 Strain Isolated in 2006 from a Patient in Shenzhen, Southern China, with a Lethal Case of Enterovirus Infection

**DOI:** 10.1128/genomeA.00074-18

**Published:** 2018-03-08

**Authors:** Xiang-Jie Yao, Long Chen, Hong Yang, Jun Meng, Hai-Long Zhang, Min Jiang, Hong-Yu Zhang, Ya-Qing He, Chun-Li Wu, Ren-Li Zhang

**Affiliations:** a Institute of Pathogen Biology, Shenzhen Center for Disease Control and Prevention, Shenzhen, China

## Abstract

The whole-genome sequence of an enterovirus A71 strain (EV71/SHENZHEN001/2006) isolated in 2006 from a patient with a fatal case of enterovirus infection was determined. Phylogenetic analysis based on the complete VP1 gene classified this strain as subgenotype C4a.

## GENOME ANNOUNCEMENT

Enterovirus A71 (EV-A71), a member of the genus Enterovirus of the family Picornaviridae, is one of the main causative agents of hand, foot, and mouth disease (HFMD) in children and infants across the Asia-Pacific region (1–4). EV-A71 infections were frequently associated with various central nervous system symptoms, including aseptic meningitis, encephalitis, acute flaccid paralysis, and even death (5–7). The large EV-A71 outbreaks resulted in hundreds of deaths in mainland China in 2008. In the same year, HFMD was designated a class C notifiable disease (8–10). Complete genome sequences of EV-A71 strains isolated before 2008 are useful for understanding the genetic evolution of EV-A71 in mainland China.

On June 20, 2006, a 2-year-old girl was diagnosed at the Children’s Hospital of Shenzhen with severe hand, foot, and mouth disease. The patient manifested fever, sparse rash on the feet and buttocks, and oral ulcers before being sent to the hospital. She then developed vomiting, lethargy, muscle twitching, and aseptic encephalitis. The girl died a few days after hospitalization. Fecal specimens from this case were positive for EV-A71, as detected by real-time reverse transcription-PCR (RT-PCR). Also, the strain was cultured in RD cell lines. Then, a pair of universal primers, EVA-F16 (5′-TTAAAACAGCCTGTGGGTTGCACCCACTC-3′) and EVA-R13 (5′-TTTTTTTTTTTTTTTTTTTTTTTGCTATTCT-3′), was designed to amplify the whole genome of the EV-A71 strain using a Primescript One Step RT-PCR kit version 2 (TaKaRa, Japan). Amplified DNA product was sequenced by a commercial corporation (TaKaRa, Japan) using a primer-walking method. Contigs were assembled using Sequencer version 4.9. The raw genome sequences were examined before submission to GenBank by using BioEdit version 7.2.5. Molecular phylogeny was investigated using the program MEGA 6.06 (11).

The full-length genome of the EV-A71 strain EV71/SHENZHEN001/2006 was composed of 7,407 nucleotides (nt), excluding the poly(A) tail. The 5′ untranscribed region (UTR) was found to be 743 nt, followed by an open reading frame (ORF) encoding the structural protein P1 (2,586 nt), the nonstructural proteins P2 (1,734 nt) and P3 (2,259 nt), and the 3′ UTR (82 nt). The contents of A, C, G, and U were 26.98%, 24.11%, 23.97%, and 24.88%, respectively, with a G+C content of 48.09%. The genome sequence of EV71/SHENZHEN001/2006 was found to be closely related to that of the Chinese EV-A71 strain 2006529 (GenBank accession no. KP266579), with 98% nucleotide identity based on results of a BLAST search. The EV-A71 strain EV71/SHENZHEN001/2006 was assigned to subgenotype C4a based on phylogenetic analysis of the VP1 gene.

### Accession number(s).

The full-length genome sequence of the strain EV71/SHENZHEN001/2006 from this study was deposited in GenBank under the accession no. MG208882.
